# Correlates of health and healthcare performance: applying the Canadian health indicators framework at the provincial-territorial level

**DOI:** 10.1186/1472-6963-5-76

**Published:** 2005-12-01

**Authors:** OA Arah, GP Westert

**Affiliations:** 1 Department of Social Medicine, Academic Medical Center, University of Amsterdam, PO Box 22700, 1100 DE Amsterdam, the Netherlands; 2 Center for Prevention and Health Services Research, National Institute of Public Health and the Environment (RIVM), PO Box 1, 3720 BA Bilthoven, the Netherlands; 3 Tranzo, Faculty of Social and Behavioural Sciences, Tilburg University, PO Box 90153, 5000 LE Tilburg, the Netherlands

## Abstract

**Background:**

Since, at the health system level, there is little research into the possible interrelationships among the various indicators of health, healthcare performance, non-medical determinants of health, and community and health system characteristics, we conducted this study to explore such interrelationships using the Canadian Health Indicators Framework.

**Methods:**

We conducted univariate correlational analyses with health and healthcare performance as outcomes using recent Canadian data and the ten Canadian provinces and three territories as units of the analyses. For health, 6 indicators were included. Sixteen healthcare performance indicators, 12 non-medical determinants of health and 16 indicators of community and health system characteristics were also included as independent variables for the analysis. A set of decision rules was applied to guide the choice of what was considered actual and preferred performance associations.

**Results:**

Health (28%) correlates more frequently with non-medical determinants than healthcare does (12%), in the preferred direction. Better health is only correlated with better healthcare performance in 13% of the cases in the preferred direction. Better health (24%) is also more frequently correlated with community and health system characteristics than healthcare is (13%), in the preferred direction.

**Conclusion:**

Canadian health performance is a function of multiple factors, the most frequent of which may be the non-medical determinants of health and the community characteristics as against healthcare performance. The contribution of healthcare to health may be limited only to relatively small groups which stand to benefit from effective healthcare, but its overall effect may be diluted in summary measures of population health. Interpreting multidimensional, multi-indicator performance data in their proper context may be more complex than hitherto believed.

## Background

In June 2000, in its ambitious comparison of 191 countries in terms of their ability to meet three main goals – improving health, increasing responsiveness to meet the legitimate demands of the population, and ensuring that financial burdens are distributed fairly – the World Health Organization (WHO) ranked Canada 30^th ^in overall health system performance [1]. This was considered a further blow to an already shaken collective psyche in Canada [2]. Canada, which WHO also ranked 35^th ^on health level performance, has taken these rankings to be indicative of serious performance problems, despite the methodological criticisms leveled against the rankings [3]. In its letter to the then minister of health, the Canadian Medical Association called the report "a serious wake-up call" [3]. By September 2000, Canada's Prime Minister and the First Ministers had made a commitment to produce regular public reports on the performance of their health system [4].

**Figure 1:**
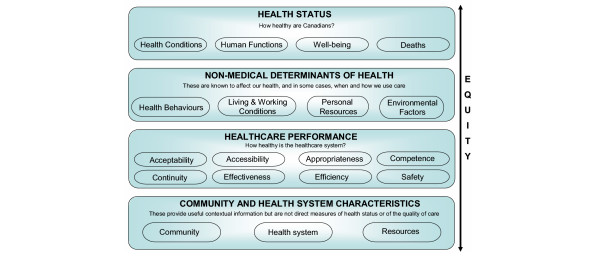
**Canadian health indicators framework **(adapted from public domain sources [12-14]). (*Legend/Footnote*: The third tier "healthcare performance" is originally titled "health system performance" in the Canadian public domain documents, refs. [12-14])

As such, the Canadian government has invested heavily in measuring and reporting on the performance of its health system at various levels [4-6]. In doing so and in line with its longstanding 'health determinants' approach to national health policy [7-9], Canada takes a broad *health performance *approach to quantifying health and healthcare progress [10]. This has entailed the development and use of a multi-dimensional 'health indicators framework' [10] (see Figure 1). This Canadian Health Indicators Framework (CHIF) has four main tiers, namely (a) health status (4 fields); (b) non-medical determinants of health (4 fields); (c) health system performance, or more appropriately referred to as *healthcare performance *in this paper, (8 dimensions or fields); and (d) community and health system characteristics (3 fields). Many of the fields or dimensions within the framework have so far been populated with indicators. The choice of a health performance framework built on the Lalonde health determinants model should come as no surprise since this was the country that introduced the Lalonde model to the world about three decades ago [9].

It is expected that the multi-dimensional framework will aid the gauging of health progress in a fair and balanced manner. Particularly, it is often assumed that the various fields are interlinked in complex ways that contribute to health and healthcare performance [11]. Healthcare functioning is also taken to be an important contributor to health, notably for those specific populations that stand to benefit from healthcare services. Yet, there has been no research into whether such links exist within the CHIF. This paper aims to examine such links. Using the CHIF as a linked model, this study analyzes the possible relations between, on the one hand, indicators of health (and healthcare performance) and, on the other hand, indicators of non-medical determinants of health, community and health system characteristics, and healthcare performance. Thus, this study poses the question whether the performance of the Canadian provinces/territories on a health (or healthcare) indicator is related to their performance on an indicator of non-medical determinants, healthcare or community characteristic. This study provides an illustrative interlinking of multi-dimensional performance at the provincial/territorial level, but not necessarily the causal interrelationships between and among indicators. We define 'health performance' as the ultimate health outcomes (measured as health status, morbidity or mortality) of a society given its mix of healthcare and non-medical determinants of health. We also define 'healthcare performance' as the degree of maintenance of healthcare system functioning (measured in terms of dimensions such as effectiveness, patient-centeredness and so forth) that is in keeping with the system's societal, professional and user goals and norms. Therefore, healthcare performance should, in principle, contribute to health performance.

## Methods

### Study population, data and measures

We used recent (2001 to 2003) secondary data on the performance of the thirteen Canadian provinces (ten in number) and territories (a total of 3) usually reported on by the government [12-16] (see Table 1). The ten provinces are Newfoundland & Labrador, Prince Edward Island, Nova Scotia, New Brunswick, Quebec, Ontario, Manitoba, Saskatchewan, Alberta, and British Columbia. The three territories comprise Yukon Territory, Northwest Territories, and Nunavut. These are the 13 jurisdictions that have constitutional responsibility for Canadian health and healthcare. The provinces and territories are of varying sizes, demographic constitution and capacities. The data underpin the annual *Health Indicator *publications which accompany the national *Health Care in Canada *reports [12,15]. The data which cover 95% of the Canadian population are appropriately age, population and gender weighted. Their primary collection sources include the Canadian Community Health Survey (in 2000/01 and 2003), Hospitality Mortality Database (CIHI), Discharge Abstract Database (CIHI) and the databases of the Centre for Infectious Disease Prevention and Control (Health Canada).

**Table 1:** Descriptive statistics for the Canadian provinces and territories, in 2001 [12-14]

**Province/territory**	**Total population**	**Elderly population **(%)	**Urban population **(%)	**Total health expenditure per capita **($)	**Life expectancy at birth **(years)
Newfoundland and Labrador (NF)	534 000	11.9	57.6	3,468	78.1
Prince Edward Island (PE)	139 000	13.4	44.5	3,324	78.9
Nova Scotia (NS)	943 000	13.5	55.6	3,208	78.9
New Brunswick (NB)	756 000	13.1	50.2	3,267	79.0
Quebec (QC)	7 418 000	13.0	80.2	3,112	79.4
Ontario (ON)	11 895 000	12.6	84.6	3,492	79.9
Manitoba (MB)	1 149 000	13.6	71.7	3,706	78.6
Saskatchewan (SK)	1 017 000	14.6	64.1	3,422	79.2
Alberta (AB)	3 059 000	10.2	80.7	3,552	79.7
British Columbia (BC)	4 102 000	13.2	84.6	3,569	80.4
Yukon Territory (YK)	30 000	5.8	58.7	4,789	77.5
Northwest Territories (NT)	41 000	4.3	58.3	6,450	77.0^†^
Nunavut (NU)	28 000	2.5	32.4	6,306	69.4
*Canada*	*31 111 0000*	*12.6*	*79.6*	*3,416*	*79.6*

^†^Pooled over 1995–97

To address the question whether provinces and territories that are better off in one indicator are also better off in the preferred direction in other indicators, we chose two main outcome tiers from the CHIF. First, for the outcome 'health performance' (health) we included 6 indicators from the *health status *tier (see Table 2): well-being (1 indicator), health conditions (3 indicators), human function (1 indicator), and deaths (1 indicator). Second, for the outcome 'healthcare performance' (healthcare) we included sixteen indicators for *healthcare performance *tier to cover acceptability, accessibilty, appropriateness, effectiveness, safety, and health surveillance dimensions. There are currently little or no routinely reported indicators for the competence, continuity and efficiency dimensions. The choice of indicators from the entire set of measures within the CHIF was guided by data availability and completeness (for at least ten provincial/territorial units). The independent variables consisted of indicators of non-medical determinants of health, community and health system characteristics, and healthcare performance. For the *non-medical determinants of health *(see Additional file 1), twelve indicators were chosen covering health behaviors, living and working conditions, personal resources and environmental factors. In addition, sixteen indicators were selected from the *community and health system characteristics *tier to cover the community, health system and resource fields. The details of these explanatory variables or indicators are available on request from the first author, and can also be found in the reference [12-16]. An indicator was considered positive (negative) if higher (lower) values of it would be **preferred **in reality. Whenever it was unclear whether a higher or lower level of an indicator would be preferred, we labeled it as both positive and negative (+/-).

**Table 2:** Health and healthcare performance indicators: descriptions, estimates, Canadian averages, and data sources [12-16]

**Indicator**	**Description**	**Provincial & Territorial value Range**	**Canadian average **(95% C.I.)	**Data source**
***Health status ***'Tier'				
• **Well-being**				
Self-rated health (excellent or very good) [+]	Percentage of the population aged 12 and over who rate their own health status as either excellent or very good	53.2–66.2%	61.4 (61.0–61.8)	Statistics Canada, CCHS 2000–01
• **Health conditions**				
Body mass index higher than 27 [-]	Body weight in kilograms divided by the squared value of the height in meters with values greater than 27 (overweight) for those aged 20 to 64	29.0–42.8 kg/m^2^	31.9 (31.4–32.3)	Statistics Canada, CCHS 2000–01
Asthma rate [-]	Percentage of the population aged 12 and over who report that they have been diagnosed by a health professional as having asthma	3.6–9.2%	8.4 (8.2–8.6)	Statistics Canada, CCHS 2000–01
Diabetes rate [-]	Percentage of the population aged 12 and over who report that they have been diagnosed by a health professional as having diabetes	1.9–5.8%	4.1 (4.0–4.3)	Statistics Canada, CCHS 2000–01
• **Human function**				
Functional health (perfect of very good) [+]	Percentage of the population aged 12 and over reporting measures of overall functional health, based on eight dimensions of functioning (vision, hearing, speech, mobility, dexterity, feelings, cognition and pain)	66.3–84.9%	80.5 (80.1–80.8)	Statistics Canada, CCHS 2000–01
• **Deaths**				
Life expectancy [+]	Life expectancy at birth calculated in years for overall population	69.4–80.4 years	79.6 (79.6–79.7)	Statistics Canada
				
***Healthcare performance ***'Tier'				
• **Acceptability**				
Satisfied with family doctor [+]	Population aged 15 and above who report being very or somewhat satisfied with the most recent family doctor or other physician care received	88.3–93.5%	91.4 (90.7–92.0)	Statistics Canada, CCHS 2003
Satisfied with health care services [+]	Population aged 15 and above who report being very or somewhat satisfied with health care services received in the past 12 months	74.2–88.6%	84.9 (84.3–85.6)	Statistics Canada, CCHS 2003
Satisfied with community health care [+]	Population aged 15 and above who report being very or somewhat satisfied with community health care received in the past 12 months	78.0–91.9%	83.0 (81.4–84.6)	Statistics Canada, CCHS 2003
• **Accessibility**				
Screening mammography [+]	Women aged 50 to 69 who reported having had a mammogram for routine screening within the past 2 years	36–54%	52	Statistics Canada, CCHS 2000/01
Pap smear [+]	Women aged 18 to 69 who reported having had a Pap smear test for routine for routine screening within the past 3 years	65–81%	73	Statistics Canada, CCHS 2000/01
Difficulties accessing routine care [-]	Population aged 15 and above who report difficulties accessing routine or on-going care, among those who required care at any time of day	12.2–20.4%	16.4 (15.3–17.5)	Statistics Canada, CCHS 2003
Difficulties accessing health information [-]	Population aged 15 and above who report difficulties accessing health information or advice, among those who required care at any time of day	12.3–17.8%	15.8 (14.7–16.9)	Statistics Canada, CCHS 2003
• **Appropriateness**				
Vaginal birth after Caesarean section [+]	Proportion of women who have previously had a caesarean section who give birth via vaginal delivery in an acute care hospital	12.5–60.7%	26.7 (26.2–27.2)	CIHI, Discharge abstract database
Caesarean sections [-]	Proportion of women delivering babies in acute care hospitals by caesarean section (stillbirths are excluded from denominator due to database characteristic)	9.2–27.9%	22.5 (22.3–22.6)	CIHI, Hospital mortality database
• **Effectiveness**				
In-hospital 30-day stroke mortality [-]	Risk-adjusted rate of all-cause in-hospital death occurring within 30 days of first admission to an acute care hospital with a diagnosis of stroke (aged 20 to 105 years)	15.5–24.2%	18.7	CIHI, Hospital mortality database
Pneumonia readmission rate [-]	Risk-adjusted rate of unplanned readmission following discharge for pneumonia (aged 15 to 84 years) within 28 days of index episode; based on three years of pooled data	2.7–6.9%	3.2	CIHI, Discharge abstract database
Ambulatory care sensitive conditions [-]	Age-standardized inpatient acute care hospitalization rate for conditions where appropriate ambulatory care prevents or reduces the need for hospitalization, per 100,000 population	243–1,114	346 (344–348)	CIHI, Hospital mortality database
Pneumonia & influenza hospitalizations [-]	Age-standardized acute care hospitalization rate for pneumonia and influenza, per 100,000 population aged 65 and older	482–2566	768 (760–777)	CIHI, Hospital mortality database
• **Safety**				
Hip fracture hospitalizations [-]	Age-standardized hospitalization rate for fracture of the hip, per 100,000 population aged 65 and older	495–660	554 (547–561)	CIHI, Hospital mortality database
• **Other: health surveillance**				
Chlamydia [-]	Number of cases of genital chlamydia reported, per 100,000 population	89.14–2514.29	149.19	CIDPC, 2000
Hepatitis C [-]	Number of cases of hepatitis C reported, per 100,000 population	9.74–156.67	60.42	CIDPC, 2000

[-] implies that lower levels of the indicator are preferred; [+] implies that higher levels of the indicator are preferred

CCHS: Canadian Community Health Survey

CIHI: Canadian Institute for Health Information

CIDPC: Centre for Infectious Disease Prevention and Control, Health Canada

### Analysis

Pearson's correlation coefficient *r *was used to estimate the univariate associations between the indicators of the 2 main outcomes (health and healthcare) and the indicators of non-medical determinants, community and health system characteristics, and healthcare performance. The unit of analysis was each of the thirteen Canadian provinces and territories. The indicators were log-transformed to avoid spurious correlations between rate-based indicators. Furthermore, given the small sample size (*N *= 13), we used critical (cut-off) values of *r *to ensure that the correlation was real and to minimize the chances of committing type I error, that is incorrectly rejecting a true statistical null hypothesis. At a significance level of 5%, the critical values of *r *for small sample sizes are as follows: r* ≥ 0.552, 0.576, 0.602 or 0.631 for *N *= 13, 12, 11, or 10 respectively. At a significance level of 1%: r* ≥ 0.683, 0.707, 0.734 or 0.764 for *N *= 13, 12, 11, or 10 respectively [17]. Another conservative statistical choice was that we used two-sided *p-*values to assess the significance of the correlations.

We applied decision rules to aid the interpretation of significant correlations. A correlation between any two indicators *i *and *j *was considered a **significant preferred **performance association if the coefficient *r *was positive when both *i *and *j *were positive or when both were negative. However, if one indicator was positive while the other was negative, the correlation between them was considered a significant preferred performance association if the coefficient *r *was negative. In all cases, the abovementioned critical value (r*) requirement must have been met. Assuming a null hypothesis H_0 _that *r *= 0, the following decision rules were applied to the univariate correlations:

(a) if indicators *i *and *j *were both either positive (+) or negative (-), then the following decision rule applied to their **preferred positive **correlation *r*_ij_:

*r *≥ r* → reject H_0_

*r *<r* → do not reject H_0_

(b) if only one of the indicators *i *and *j *was positive (+), then the following decision rule applied to their **preferred negative **correlation *r *(where r* took on a negative value):

*r *≤ r* → reject H_0_

*r *> r* → do not reject H_0_

Whenever the correlation was significant but not in the preferred direction, the result was considered a suboptimal performance association (termed in this paper as **not preferred **performance) that could point towards possibilities for improvement. To estimate the uncertainty around each estimated *r*, we wrote a simple spreadsheet for calculating the 95% confidence interval (C.I.) for each sample size. (This spreadsheet is available on request from the first author.)

As far as the decision rules were concerned, caution was exercised in applying them to indicators for which it was unclear whether higher or lower values would be preferred. For this reason, the decision rules were not applied to three indicators of community characteristics, namely population size, elderly population, and urban population.

All analyses were carried out using SPSS version 12.0.2 (SPSS Inc., Chicago, IL) and Microsoft^® ^Excel 2002 SP3 (Microsoft Corporation, Redmond, WA).

## Results

Table 3 shows that significant 'preferred' correlations between health and non-medical determinants of health range from -0.853 (95% C.I.: -0.955 to -0.570; *P *< 0.05) for the association between body mass index and dietary practices to 0.836 (95% C.I.: 0.528 to 0.950; *P *< 0.01) for the association between unemployment rate and diabetes rate. Smoking status, having high proportions of high school and post-secondary graduates displayed unfavorable ('not preferred') associations with health indicators (Table 3).

**Table 3:** Correlations between health (status) indicators and non-medical determinants of health

	**Self-rated health (excellent or very good) **[+]	**Body mass index higher than 27 **[-]	**Asthma rate **[-]	**Diabetes rate **[-]	**Functional health (perfect or very good) **[+]	**Life expectancy **[+]
***Non-medical determinants of health***						
**Health behaviors**						
Smoking status [-]	**-0.586**^†^	*0.337*	-0.688^†^	-0.794^‡^	**-0.583**^†^	**-0.633**^‡^
Frequency of heavy drinking [-]	*-0.391*	**0.571**^†^	-0.252	-0.437	*-0.161*	**-0.783**^‡^
Leisure-time physical activity [+]	*0.121*	**-0.600**^†^	0.079	*-0.339*	*0.116*	**0.710**^†^
Dietary practices [+]	*0.420*	**-0.853**^†^	0.258	0.135	*0.269*	**0.633**^†^
**Living and working conditions**						
High school graduates [+]	*0.490*	*-0.485*	0.855^‡^	0.622^†^	**0.712**^‡^	**0.754**^†^
Post-secondary graduates [+]	*0.451*	*-0.456*	0.711^‡^	0.237	**0.556**^†^	*0.585*
Unemployment rate [-]	0.524	*0.546*	-0.497	**0.836**^‡^	0.432	**-0.727**^†^
Youth unemployment [-]	0.396	*0.398*	-0.436	**0.791**^‡^	0.260	*-0.284*
Low income rate [-]	*-0.050*	-0.143	-0.419	*0.145*	0.556^†^	*-0.196*
Average personal income [+]	-0.203	*-0.411*	0.207	**-0.546**^†^	*0.063*	**0.821**^‡^
**Personal resources**						
Life stress [-]	*-0.008*	-0.468	**0.652**^†^	*0.177*	0.516	0.581
**Environmental factors**						
Exposure to second-hand smoke [-]	*-0.252*	**0.590**^†^	*0.083*	-0.071	0.238	**-0.676**^†^

[-] implies that lower levels of the indicator are preferred

[+] implies that higher levels of the indicator are preferred

^† ^P < 0.05

^‡ ^P < 0.01

**Bold**: correlation is significant in the possibly preferred direction and exceeds the critical level necessary for the sample size

*Italicized*: correlation is in the possibly preferred direction but is *not *significant at the critical level necessary for the sample size

Table 4 shows the correlations between health and healthcare performance indicators. Here, the significant 'preferred' correlations range from -0.782 (95% C.I.: -0.932 to -0.406; *P *< 0.01) for the association between provincial/territorial performance on diabetes rate and vaginal birth after Caesarean section to 0.754 (95% C.I.: 0.347 to 0.922; *P *< 0.01) for the association between provincial/territorial performance on diabetes rate and in-hospital 30-day stroke mortality. Table 5 shows the correlations of health indicators with community and health system characteristics. Again, significant 'preferred' correlations range from -0.893 (95% C.I.: -0.968 to -0.673; *P *< 0.01) for the association between knee replacement and functional health status (as perfect or very good) to 0.810 (95% C.I.: 0.468 to 0.941; *P *< 0.01) for the association between provincial/territorial performance on diabetes rate and hysterectomy. Likewise, several 'not preferred' performance associations exist between health indicators and mostly (health system) resource indicators (Table 5).

**Table 4:** Correlations between health (status) indicators and healthcare performance indicators

	**Self-rated health (excellent or very good) **[+]	**Body mass index higher than 27 **[-]	**Asthma rate **[-]	**Diabetes rate **[-]	**Functional health (perfect or very good) **[+]	**Life expectancy at birth **[+]
***Healthcare performance***						
**Acceptability**						
Satisfied with family doctor [+]	*0.300*	0.356	0.360	0.788^‡^	*0.440*	-0.605
Satisfied with health care services [+]	*0.479*	*-0.012*	0.756^‡^	0.727^‡^	*0.708*	-0.234
Satisfied with community health care [+]	-0.170	0.367	-0.445	0.055	-0.073	-0.500
**Accessibility**						
Screening mammography [+]	*0.168*	*-0.362*	*-0.025*	0.247	-0.266	**0.668**^†^
Pap smear [+]	*0.275*	0.346	0.622^†^	0.488	*0.403*	*0.470*
Difficulties accessing routine care [-]	0.595	*0.248*	-0.103	*0.447*	0.760^†^	**-0.657**^†^
Difficulties accessing health information [-]	0.141	-0.074	*0.188*	*0.277*	0.021	*-0.049*
**Appropriateness**						
Vaginal birth after Caesarean section [+]	-0.416	*-0.197*	**-0.671**^†^	**-0.782**^‡^	-0.770^†^	*0.309*
Caesarean sections [-]	0.511	-0.034	**0.700**^†^	**0.706**^‡^	0.667^†^	*-0.005*
**Effectiveness**						
In-hospital 30-day stroke mortality [-]	0.273	**0.687**^†^	-0.381	**0.754**^‡^	0.079	**-0.754**^†^
Pneumonia readmission rate [-]	0.190	-0.524	*0.406*	-0.347	0.112	0.307
Ambulatory care sensitive conditions [-]	*-0.112*	*0.467*	*0.304*	*0.016*	0.308	*-0.392*
Pneumonia & influenza hospitalizations [-]	*-0.434*	*0.502*	-0.089	-0.380	*-0.153*	*-0.410*
**Safety**						
Hip fracture hospitalizations [-]	*-0.305*	*0.070*	-0.190	-0.615^†^	*-0.153*	0.398
**Other: health surveillance**						
Chlamydia [-]	**-0.661**^†^	*0.216*	-0.818^‡^	-0.869^‡^	**-0.776**^‡^	*-0.061*
Hepatitis C [-]	*-0.082*	-0.669^†^	*0.309*	-0.321	0.212	0.823^‡^

[-] implies that lower levels of the indicator are preferred

[+] implies that higher levels of the indicator are preferred

^† ^P < 0.05

^‡ ^P < 0.01

**Bold**: correlation is significant in the possibly preferred direction and exceeds the critical level necessary for the sample size

*Italicized*: correlation is in the possibly preferred direction but is *not *significant at the critical level necessary for the sample size

**Table 5:** Correlations between health (status) indicators and community & health system characteristics

	**Self-rated health (excellent or very good) **[+]	**Body mass index higher than 27 **[-]	**Asthma rate **[-]	**Diabetes rate **[-]	**Functional health (perfect or very good) **[+]	**Life expectancy at birth **[+]
***Community & health system characteristics***						
**Community**^¥^						
Population [+/-]	0.308	-0.552	0.221	0.125	0.239	0.620
Elderly population [+/-]	0.461	-0.084	0.512	0.841	0.449	0.780^‡^
Dependency ratio [-]	*-0.525*	*0.252*	-0.739^‡^	-0.497	**-0.814**^‡^	**-0.638**^†^
Urban population [+/-]	0.350	-0.646^†^	0.528	0.213	0.518	0.708^†^
**Health system**						
Hip replacement [-]	*-0.105*	-0.092	*0.243*	-0.065	*-0.311*	0.196
Knee replacement [-]	**-0.652**^‡^	*0.228*	-0.628^†^	-0.749^‡^	**-0.893**^‡^	**-0.774**^‡^
Hysterectomy [-]	0.396	*0.252*	*0.459*	**0.810**^‡^	0.356	0.460
Bypass surgery [-]	0.634^†^	*0.236*	-0.029	**0.584**^‡^	0.155	**-0.701**^†^
**Resources**						
Total health expenditure per capita [+]	-0.467	0.068	**-0.727**^‡^	**-0.814**^‡^	-0.694^‡^	-0.773^‡^
Public sector health expenditure per capita [+]	-0.473	0.084	*-0.544*	**-0.807**^‡^	-0.510	-0.417
General/family physicians [+]	*0.504*	*-0.416*	0.645^‡^	0.436	**0.641**^‡^	**0.575**^†^
Certified specialists [+]	*0.399*	*-0.280*	0.556^†^	0.626^†^	*0.499*	**0.760**^‡^
Registered nurses [+]	-0.310	0.738^‡^	*-0.175*	*-0.122*	*0.011*	-0.435
Licensed practical nurses [+]	*0.496*	0.663^‡^	*-0.386*	0.756^‡^	*0.206*	-0.336
Pharmacists [+]	*0.546*	0.010	0.798^‡^	0.646^‡^	**0.728**^‡^	**0.782**^‡^
Total physicians [+]	**0.576**^†^	*-0.445*	0.772^‡^	0.696^‡^	**0.730**^‡^	**0.872**^‡^

[-] implies that lower levels of the indicator are preferred

[+] implies that higher levels of the indicator are preferred

^† ^P < 0.05

^‡ ^P < 0.01

**Bold**: correlation is significant in the possibly preferred direction and exceeds the critical level necessary for the sample size

*Italicized*: correlation is in the possibly preferred direction but is *not *significant at the critical level necessary for the sample size

^¥ ^Only the dependency ratio indicator is assessed here using the decision rule since the other indicators could be preferred either way depending on the goal and audience

The table in Additional file 2 gives an overview of the correlations between healthcare performance, on the one hand, and non-medical determinants of health and community and health system characteristics, on the other hand. The significant 'preferred' correlations range from -0.863 (95% C.I.: -0.958 to -0.595; *P *< 0.01) for the association between provincial/territorial performance on screening mammography and its frequency of heavy drinking to 0.944 (95% C.I.: 0.819 to 0.983; *P *< 0.01) for the performance association between smoking status and chlamydia cases per unit population. Several 'not preferred' associations also exist for the healthcare performance outcome. For instance, the share of public sector health expenditure per capita shows several strong correlations with healthcare indicators: ranging from -0.716 (95% C.I.: -0.909 to -0.273; *P *< 0.01) for being satisfied with family doctor to 0.851 (95% C.I.: 0.565 to 0.954; *P *< 0.01) for chlamydia cases per unit population. Preferred inter-correlations among healthcare performance indicators ranged from -0.867 (95% C.I.: -0.960 to -0.605; *P *< 0.01) for Caesarean section rate versus vaginal birth after Caesarean section to 0.716 (95% C.I.: 0.273 to 0.909; *P *< 0.05) for the association between being satisfied with family doctor and being satisfied with healthcare services.

Tables 6 and 7 present the summaries of the correlates of health and healthcare performance. Table 6 shows that there are relatively more 'preferred' associations between health and non-medical determinants (that is, 20 out of 72 correlations, or 28%) than between health and healthcare performance indicators (that is, 12 out of 96 correlations, or almost 13%). Similarly, there are relatively fewer 'not preferred' correlations between health and non-medical determinants (that is, 6 out of 72 correlations, or 8%) than between health and healthcare performance indicators (that is, 11 out of 96 correlations, or 11%). Also, the associations between health and community/health system characteristics out-number those between health and healthcare performance. There are 19 significant 'preferred' associations out of 78 correlations between health and community/health system characteristics (that is, almost 24%), while there are 18 significant 'not preferred' associations (that is, 23%). Table 7 shows that out of 190 correlations between healthcare and non-medical determinants, the significant 'preferred' and 'not preferred' associations are respectively 23 (12%) and 18 (9%). Based on 208 correlations between healthcare and community/health system characteristics, there are 27 (13%) and 35 (17%) significant 'preferred' and 'not preferred' associations respectively. Interrelationships among healthcare indicators are few in number within and between dimensions (details not shown but summarized in Table 7; see Additional file 2).

**Table 6:** Summary of significant 'preferred' and 'not preferred' performance correlates of health (status) indicators^†^

	**Self-rated health (excellent or very good)**	**Body mass index higher than 27**	**Asthma rate**	**Diabetes rate**	**Functional health (perfect or very good)**	**Life expectancy**	*Row Total*^¥^
***Non-medical determinants of health ***(number of indicators)							
Health behaviors (4)	1/0	3/0	0/1	0/1	1/0	4/0	9/2/24
Living and working conditions (6)	0/0	0/0	0/2	3/1	2/1	3/0	8/4/36
Personal resources (1)	0/0	0/0	1/0	0/0	0/0	0/0	1/0/6
Environmental factors (1)	0/0	1/0	0/0	0/0	0/0	1/0	2/0/6
*Sub-Total*							*20/6/72*
***Healthcare performance ***(number of indicators)							
Acceptability (3)	0/0	0/0	0/1	0/2	0/0	0/0	0/3/18
Accessibility (4)	0/0	0/0	0/1	0/0	0/1	2/0	2/2/24
Appropriateness (2)	0/0	0/0	2/0	2/0	0/2	0/0	4/2/12
Effectiveness (4)	0/0	1/0	0/0	1/0	0/0	1/0	3/0/24
Safety (1)	0/0	0/0	0/0	0/1	0/0	0/0	0/1/6
Other: health surveillance(2)	1/0	0/1	1/0	0/1	1/0	0/1	3/3/12
*Sub-Total*							*12/11/96*
***Community and health system characteristics ***(number of indicators)							
Community (1)^‡^	0/0	0/0	0/1	0/0	1/0	1/0	2/1/6
Health system (4)	1/1	0/0	0/1	2/1	1/0	2/0	6/3/24
Resources (8)	1/0	0/2	1/4	2/4	3/1	4/3	11/14/48
*Sub-Total*							*19/18/78*
*Column Total*	*4/1*	*5/3*	*5/11*	*10/11*	*9/5*	*18/4*	-

^†^Numbers (x/y) in cells respectively represent the number of indicators which showed significant **preferred **performance association (x) and the number of indicators which showed significant **not preferred **performance association (y) at the provincial/territorial level

^¥^Total number of significant **preferred **correlations/total number of significant **not preferred **correlations/total number of tested correlations for that performance dimension or indicator group

^‡^Only the dependency ratio indicator is assessed here using the decision rule since the other indicators could be preferred either way depending on the goal and audience

**Table 7:** Summary of significant 'preferred' and 'not preferred' correlates of healthcare performance indicators^†^

	**Acceptability **(3)	**Accessibility **(4)	**Appropriateness **(2)	**Effectiveness **(4)	**Safety **(1)	**Other: health surveillance **(2)	*Row Total*^¥^
***Non-medical determinants of health ***(number of indicators)							
Health behaviors(4)	1/1	3/0	0/2	4/1	0/0	2/1	10/5/64
Living and working conditions (6)	1/3	4/1	3/3	2/1	0/1	1/4	11/13/96
Personal resources (1)	0/0	1/0	0/0	0/0	0/0	0/0	1/0/16
Environmental factors (1)	0/0	0/0	0/0	1/0	0/0	0/0	1/0/16
*Sub-Total*							*23/18/190*
***Community and health system characteristics ***(number of indicators)							
Community (1)^‡^	0/0	1/1	0/2	0/0	0/0	1/0	2/3/16
Health system (4)	3/2	0/2	1/2	0/0	0/1	1/1	6/8/64
Resources (8)	4/4	2/3	3/6	3/7	2/1	5/3	19/24/128
*Sub-Total*							*27/35/208*
***Healthcare performance ***(number of indicators)							
Acceptability (3)	-	0/0	0/1	0/1	1/0	2/0	-
Accessibility (4)	-	-	0/2	2/0	0/0	1/1	-
Appropriateness (2)	-	-	-	0/0	0/0	0/2	-
Effectiveness (4)	-	-	-	-	2/0	1/1	-
Safety (1)	-	-	-	-	-	1/0	-
Other: health surveillance (2)	-	-	-	-	-	-	-
*Column Total*	*9/10*	*11/7*	*7/18*	*12/10*	*5/3*	*15/13*	-

^†^Numbers (x/y) in cells respectively represent the number of indicators which showed significant **preferred **performance association (x) and the number of indicators which showed significant **not preferred **performance association (y) at the provincial/territorial level

^¥ ^Total number of significant **preferred **correlations/total number of significant **not preferred **correlations/total number of tested correlations for that performance dimension or indicator group

^‡^Only the dependency ratio indicator is assessed here using the decision rule since the other indicators could be judged either way depending on the audience

-; not applied

## Discussion

This is the first study to estimate the correlates of health and healthcare performance of Canadian provinces/territories. It suggests that relatively better performance on non-medical determinants of health is related to better health. Healthcare performance is, however, less frequently related to health. In addition, health is relatively better associated with community/health system characteristics than healthcare performance is.

Provincial/territorial healthcare performance shows relatively more 'preferred' than 'not preferred' associations with non-medical determinants. Healthcare correlations with community and health system characteristics show the reverse picture, with relatively more 'not preferred' associations than 'preferred associations'. This again suggests there is still more to be desired in how provinces/territories simultaneously optimize their performance in terms of health and healthcare, given their community and health system characteristics. Interrelationships between healthcare performance indicators suggest that how the healthcare system performs in terms of one indicator is often not related to its performance in terms of another indicator. Importantly, there are at least two ways of looking at the correlations. First, the correlations can be interpreted as possible associations between the epidemiological factors that underlie the indicators (that is, *epidemiological associations*) in ideal circumstances; for example, body mass index is associated with dietary practices in an epidemiological sense. Second, the correlations could be viewed as no more than associations between the actual performance attainments of provinces/territories in terms of different indicators (that is, *performance associations*) in everyday circumstances. Although both views are related and implied in this study, we recognize that the latter can undermine the former when real "epidemiological associations" are not observed in sub-optimal or "not preferred" performance scenarios.

### Explanation of results

Health is a function of multiple factors or determinants that work in complex, sometimes unclear ways [7-9,18]. The results of this study, although based on multiple univariate correlations, support this notion. In a similar correlational analysis used in a study of 311 local administrative units covering 70 million populations in Japan, a group of nine health determinant indices (namely, healthcare resources, preventive health activities, environmental quality, housing urban clutter, local economy, employment, income, and education) explained almost 52% of the variances in health index levels in the cities studied [19]. In our study, it could be shown that, if independently assessed, non-medical determinants could explain between 40% and 67% (calculated from *r*-squared) of the variance in life expectancy as a measure of health at the provincial/territorial level (see Table 3). Similarly, healthcare performance indicators could independently account for 44% to 57% of the variance in life expectancy.

Unsurprisingly, health has relatively more associations with non-medical determinants than with healthcare indicators. Studies looking at healthcare inputs and resources to explain variations in the health of countries or other ecological units of analysis have mostly failed to demonstrate any or consistent associations [20-22]. However, it is also possible that the non-medical determinants correlate more frequently with health levels because they represent factors (such as dietary practices, smoking and so on) that are more or less related to disease risk profiles, prevalence and incidence in a general population. Healthcare factors reflect mostly corrective or management measures that marginally influence the prevalence or incidence of chronic ill-health or diseases, particularly in the face of co-morbidities in risk populations. This does not imply that healthcare is not life-saving for those who need it, when they need it. The point is that the contribution of healthcare to the general health of a population may be limited only to relatively small groups, in time and space, which stand to benefit from effective healthcare, but its overall effect will be diluted in summary measures of population health or well-being. Therefore, using the prevalence of diagnosed health conditions such as diabetes rates as indicators of health performance can only yield disappointing results in relation to healthcare performance. This explanation may be relevant to the results detailed in Table 4 when compared to Table 3 findings.

There are also results (the significant 'not preferred' correlations) which suggest that provinces may be struggling with optimizing their health performance given their health determinants, and healthcare and community profiles. For example, the higher the percentage of high school or post-secondary graduates in a province/territory, the higher the asthma rate (*r *= 0.855 or 0.711, *P *< 0.01, in Table 3). This may be expected given that asthma is more prevalent among the younger age groups. The comparable frequency with which health displays both 'preferred' and 'not preferred' associations with community and health system characteristics also points to the possibility that health levels are shaped community needs in complex ways that this study cannot disentangle.

Moreover, it is difficult to say which indicator precedes the other in this study. Healthcare indicators may just be current responses to perceived previous shortcomings in health. For instance, the negative correlation (*r *= -0.773, *P *< 0.01, in Table 5) between health expenditure and life expectancy could be due to an increase in total healthcare spending in provinces/territories with a long history of lower health levels. Nunavut, for example, is a collection of 26 communities with 28,000 inhabitants living in a vast territory about one-fifth the size of Canada. Nunavut is only accessible by air or sea, and has substantial difficulties in recruiting and retaining health professionals although it spends twice as much as the Canadian average on health per capita (Table 1). Given, the poorer-than-Canadian-average health in Nunavut, the government has been investing a lot in health and healthcare there. Therefore, health expenditure will understandably display a negative association with life expectancy.

In this study, we assumed that the lower rates of knee replacement or bypass surgery and other contextual health system indicators, when seen in the context of higher health outcomes, will be preferred. It could, however, be argued that lower rates of such contextual indicators could be indicative of unmet needs (locally). Given such interpretations, we would have to reverse the interpretations and associated correlations to reflect the possibility of under-use of needed healthcare in such communities. Nonetheless, our current interpretations allow for the possibilities of over-use of appropriate healthcare in communities where health outcomes are already high. These considerations further highlight the often overlooked difficulties that are inherent in understanding published performance data, regardless of the amount of contextual information provided. In a sense, the meaning and excellence of performance may be in the eye of the beholder.

### Implications

Recommending policies on health and healthcare in the provinces and territories must take into account the responsibility structure, organization, delivery and funding of healthcare in the concerned areas. Blanket recommendations will probably miss the point by being too generic and insensitive to local needs. The Canadian healthcare system is mainly publicly funded (Medicare) [4]. The provinces and territories have primary constitutional responsibility for health, and the management and delivery of health services, although they all adhere to a set of federal principles in view of the Canadian history of fiscal transfers from the federal to the provincial governments [23]. A number of interlocking general revenue-financed health insurance plans cover hospital in-patient and out-patient services, pharmaceutical products, physician services and public health services. Therefore, there is, in principle, a lot of improvement leverage points that provincial, territorial and federal governments can use to better the health of Canadians. Nevertheless, there are serious challenges and tensions posed by the varying needs of multiple stakeholders and the use of broad summary indicators or a parsimonious set of indicators. It is advisable that governments invest more in investigating and interpreting possible linkages among performance results in order to aid learning and to facilitate the simultaneous optimization of different performance dimensions and indicators.

That said, it is also becoming increasingly clearer that investing in public health, especially in promoting healthy life style, disease prevention and health protection, may still offer new avenues for dealing with population health in western societies [24-26]. The current narrow focus on technical care may not be the best approach to improving and maintaining population health when most gains are to be made by living and working well, for example. The social choice arrangements needed to achieve better health for Yukon Territory, Northwest Territories and Nunavut will be have to be ambitious. There are signs that some provinces are already investing more in both health and healthcare performance of their communities [27-29].

### Study limitations

The data used in this study come from multiple sources with different data elements and quality. Although the Canadian government continues to invest in the quality and coverage of the data used in constructing the indicators, the system is still not perfect [16]. To ensure data quality and comparability in performance reporting, the Canadian First Ministers have been giving policy support to the federal government and the 13 provincial/territorial jurisdictions since September 2000 [4,16]. In February 2003, the First Ministers' Accord on Health Care Renewal directed Health Ministers to further develop indicators to supplement the work on comparable indicator reporting. So far, about 70 indicators have been standardized for comparable reporting at the provincial/territorial and federal government levels.

Furthermore, it is beyond the scope of this study to ascertain which indicator is really a cause or an effect. Correlation does not imply causality, and this is troublesome in ecological observational study designs [30]. Bearing this in mind, we only hope to speak to the (sub)optimization of a pair of indicators based on the attained performance. We also realize that statistical significance does not necessarily imply substantive significance of performance. Unconfounded associations, temporality, and real-world translation of the performance associations, particularly causal ones, are more appropriate criteria for assessing importance of the association. Such assessments will also entail value judgments pertaining to how good the performance levels may be. Besides, it is quite possible there are important lag effects of health determinants and other indicators on health and healthcare performance that this study will be unable to pick up, given its contemporaneous cross-sectional ecological design.

A vexing limitation is the issue of multiple correlations and significance testing, given also that the health and healthcare indicators are not independent. At a significance level of 5%, there is a 1 in 20 chance of getting a spurious significance, just by chance. Thus, given the large number of correlations conducted in this study, it is quite likely that some correlations occurred by mere chance. However, given the rather low to moderate number of significant results and the fact that we actually pre-specified our paired analyses, it is likely that the magnitude of errors introduced by the multiple independent correlations using the same sample file will be relatively minimal. Besides, our decision rules were rather conservative. We could have set a stricter significance level by, say, dividing 0.05 by the number of anticipated tests (that is, using the Bonferroni method) [31]. Although this would minimize our type I error rate, it would have depleted our statistical power, thus giving a higher probability of type II error (that is, the probability of rejecting an association that actually exists). A trade-off between committing type I error and having enough statistical power was thus necessary, particularly given our already small sample size.

## Conclusion

The results of this exploratory study should serve as a provocative basis for future research into performance interrelationships. The prevailing assumption that publishing a comprehensive battery of indicators will automatically lead to clearer understanding and contextualization of performance is not tenable. This study forces us to take a closer look at how we actually interpret such indicators in relation to one another, and we have seen that there are no easy rules for understanding possible links between what a community attains in one indicator and what it achieves in another. Since performance is interventionist in nature, health and healthcare performance can be influenced by those who have the ability and resources to do so. This study suggests that indicators which are correlated with how well Canadian provinces and territories perform in terms of health and healthcare can act as leverage points for improving health. This study has many implications for further research on linkages within performance frameworks now being used in several industrialized countries, and for choosing a national performance framework. For instance, a framework that focuses mostly on healthcare performance (e.g. the US National Healthcare Quality Report framework or the now old UK NHS performance assessment framework) does so at the expense of understanding the links between non-healthcare determinants and population health. Further elucidation of the meanings, nature, and extent of the interrelationships among the different fields and domains of the Canadian or any other performance framework will aid actual performance improvement by pointing the responsible governments in the right direction.

## Competing interests

The author(s) declare that they have no competing interests.

## Authors' contributions

OAA conceived and supervised the study, collected the data, completed the analyses, and led the writing. GPW assisted with the study, interpretation of results and critical review of the manuscript for intellectual content.

## Pre-publication history

The pre-publication history for this paper can be accessed here:



## Supplementary Material

Additional file 1 **Canadian indicators of non-medical determinants of health **(Table in Word format; describing the non-medical determinants of health used in this study) Click here for file

Additional file 2 **Correlations between healthcare performance indicators^§^, and non-medical determinants of health and community & healthcare system characteristics **(Table in Word format; displaying extensively the correlation coefficients between healthcare performance indicators, on the one hand, and non-medical determinants of health, community characteristics and health system characteristics, on the other hand) Click here for file

## References

[B1] World Health Organization (2000). The world health report 2000 Health systems: improving performance.

[B2] Lewis S, Donaldson C, Mitton C, Currie G (2001). The future of health care in Canada. BMJ.

[B3] Deber R (2004). Why did the World Health Organization rate Canada 30th? Some thoughts on league tables. Longwoods Review.

[B4] Arah OA, Klazinga NS, Delnoij DMJ, ten Asbroek AHA, Custers T (2003). Conceptual frameworks for health systems performance: a quest for effectiveness, quality and improvement. Int J Qual Health Care.

[B5] Canadian Institute for Health Information, Statistics Canada (2000). Canadian health information roadmap initiative indicators framework.

[B6] Commission on the Future of Health are in Canada (Roy J. Romanow commissioner) (2002). Building on values: the future of health care in Canada Final report.

[B7] Evans RG, Stoddart GL (1990). Producing health, consuming health care. Soc Sci Med.

[B8] Evans RG, Stoddart GL (2003). Consuming research, producing policy?. Am J Public Health.

[B9] Lalonde M (1974). A new perspective on the health of Canadians.

[B10] Arah OA, Westert GP (2004). Perspectives on conceptualization of health and healthcare systems performance.

[B11] International Organization for Standardization (2003). Health informatics – health indicators definitions, relationships and attributes ISO TC 215/SC N339.

[B12] Canadian Institute for Health Information (2004). Health indicators 2004.

[B13] Canadian Institute for Health Information (2003). Health indicators 2003.

[B14] Canadian Institute for Health Information (2002). Health indicators 2002.

[B15] Canadian Institute for Health Information Health indicators. http://secure.cihi.ca/cihiweb/dispPage.jsp?cw_page=indicators_e.

[B16] Health Canada (2004). Healthy Canadians A federal report on comparable health indicators 2004.

[B17] Altman DG (1991). Practical statistics for medical research.

[B18] Kindig D, Stoddart G (2003). What is population health?. Am J Public Health.

[B19] Takano T, Nakamura K (2001). An analysis of health levels and various indicators of urban environments for Healthy Cities projects. J Epidemiol Community Health.

[B20] Nolte E, McKee M (2004). Does healthcare save lives? Avoidable mortality revisited.

[B21] Mackenbach JP (1991). Health care expenditure and mortality from amenable conditions in the European community. Health Policy.

[B22] Carr-Hill RA, Hardman GF, Russell IT (1987). Variations in avoidable mortality and variations in heaalth care resources. Lancet.

[B23] Wolfson M, Alvarez R, Smith P (2002). Towards integrated and coherent health information systems for performance monitoring: the Canadian experience. Measuring up: Improving health system performance in OECD countries.

[B24] World Health Organization (2002). The world health report 2002 Reducing risks, promoting healthy life.

[B25] Arah OA (2005). Performance reexamined. Concepts, content and practice of measuring health system performance. PhD thesis.

[B26] Arah OA, Westert GP, Delnoij DM, Klazinga NS (2005). Health system outcomes and determinants amenable to Public Health in industrialized countries: a pooled, cross-sectional time series analysis. BMC Public Health.

[B27] Ontario Ministry of Health and Long-Term Care (2004). Ontario's health system performance report 2004.

[B28] Statistics Canada Federal, provincial and territorial reports on comparable health indicators. http://www.statcan.ca/english/freepub/82-401-XIE/2002000/reports.htm.

[B29] Greenberg A, Angus H, Sullivan T, Brown AD (2005). Development of a set of strategy-based system-level cancer care performance indicators in Ontario, Canada. Int J Qual Health Care.

[B30] Morgenstern H, Rothman KJ, Greenland S (1998). Ecologic studies. Modern epidemiology.

[B31] Bland JM, Altman DG (1995). Statistics notes: Multiple significance tests: the Bonferroni method. BMJ.

