# Supplementary material for: Correlates of health and healthcare performance: applying the Canadian health indicators framework at the provincial-territorial level
Source: BMC Health Serv Res. 2005 Dec 1;5:76. doi: 10.1186/1472-6963-5-76 (PMC1325226; doi:10.1186/1472-6963-5-76)
Supplement: Additional file 1 — Canadian indicators of non-medical determinants of health (Table in Word format; describing the non-medical determinants of health used in this study) [file 1472-6963-5-76-S1.doc]

**Additional file 1: Canadian indicators of non-medical determinants of health**

| **Indicator** | **Description** |
| --- | --- |
| **Health behaviors** |  |
| Smoking status | Population aged 12 and over who reported being either a smoker (daily or occasional) or a non-smoker (former or never smoked) |
| Frequency of heavy drinking | Population aged 12 and over who are current drinkers and who reported drinking 5 or more drinks on at least one occasion in the past 12 months |
| Leisure-time physical activity | Population aged 12 and over reporting level of physical activity, based on their responses to questions about the frequency, duration and intensity of their participation in leisure-time physical activity |
| Dietary practices | Population aged 12 and over, by the average number of times per day that they consume fruits and vegetables |
| **Living and working conditions** |  |
| High school graduates | Population aged 25 to 29 who have a high school graduation certificate |
| Post-secondary graduates | Population aged 25 to 54 who have obtained a post-secondary certificate, diploma, or degree |
| Unemployment rate | Labour force aged 15 and over who did not have a job during the reference period |
| Youth unemployment | Labour force aged 15 to 24 years who did not have a job during the reference period |
| Low income rate | Population in economic families and unattached individuals with incomes below the Statistics Canada low-income cut-off (LICO). The cut-offs represent levels of income where people spend disproportionate amounts of money for food, shelter, and clothing |
| Average personal income | Average personal income (pre-tax, post-transfer) for persons aged 15 and over who reported income |
| **Personal resources** |  |
| Life stress | Level of chronic stress reported by the population aged 18 and over, based on their responses to a series of 17 questions about their personal situation. Here, a score of 0 or 1 was low life stress, 2 to 4 was moderate life stress and 5 to 16 was high life stress |
| **Environmental factors** |  |
| Exposure to second-hand smoke | Non-smoking population aged 12 and over who were exposed to second-hand smoke on most days in the month preceding the survey |
