# Supplementary material for: Correlates of health and healthcare performance: applying the Canadian health indicators framework at the provincial-territorial level
Source: BMC Health Serv Res. 2005 Dec 1;5:76. doi: 10.1186/1472-6963-5-76 (PMC1325226; doi:10.1186/1472-6963-5-76)
Supplement: Additional file 2 — Correlations between healthcare performance indicators§, and non-medical determinants of health and community & healthcare system characteristics (Table in Word format; displaying extensively the correlation coefficients between healthcare performance indicators, on the one hand, and non-medical determinants of health, community characteristics and health system characteristics, on the other hand) [file 1472-6963-5-76-S2.doc]

**Additional file 2: Correlations between healthcare performance indicators**^§^**, and non-medical determinants of health and community & health system characteristics**

| § | **A** [+] | **B** [+] | **C**  [+] | **D** [+] | **E** [+] | **F** [-] | **G** [-] | **H** [+] | **I** [-] | **J** [-] | **K** [-] | **L** [-] | **M** [-] | **N** [-] | **O** [**-**] | **P** [-] |
| --- | --- | --- | --- | --- | --- | --- | --- | --- | --- | --- | --- | --- | --- | --- | --- | --- |
| ***Non-Medical Determinants*** |  |  |  |  |  |  |  |  |  |  |  |  |  |  |  |  |
| **Health behaviors** |  |  |  |  |  |  |  |  |  |  |  |  |  |  |  |  |
| Smoking status [-] | *-0.494* | **-0.655**^†^ | 0.405 | **-0.731**^‡^ | *-0.326* | *0.480* | -0.453 | 0.589^†^ | -0.614^†^ | -0.118 | *0.070* | *0.208* | **0.606**^†^ | *0.467* | **0.944**^‡^ | -0.112 |
| Frequency of heavy drinking [-] | *-0.327* | *-0.393* | 0.277 | **-0.863**^‡^ | 0.294 | *0.171* | -0.249 | 0.042 | -0.091 | *0.063* | *0.046* | **0.599**^†^ | **0.878**^‡^ | *0.574* | **0.574**^†^ | *0.044* |
| Leisure-time physical activity [+] | -0.592^†^ | -0.102 | -0.395 | -0.125 | *0.171* | **-0.739**^†^ | *-0.420* | *0.322* | 0.083 | *-0.598* | 0.807^‡^ | *-0.223* | 0.034 | 0.322 | 0.023 | 0.819^‡^ |
| Dietary practices [+] | -0.014 | *0.275* | -0.077 | *0.401* | -0.289 | 0.069 | 0.181 | 0.062 | 0.162 | *-0.256* | 0.552 | *-0.499* | **-0.605**^†^ | *-0.370* | *-0.355* | 0.357 |
| **Living and working conditions** |  |  |  |  |  |  |  |  |  |  |  |  |  |  |  |  |
| High school graduates [+] | *0.259* | **0.656**^†^ | -0.363 | *0.327* | *0.429* | *-0.370* | 0.192 | -0.650^†^ | 0.727^‡^ | *-0.260* | 0.274 | *-0.086* | *-0.402* | *-0.254* | **-0.866**^‡^ | 0.399 |
| Post-secondary graduates [+] | -0.140 | *0.374* | -0.444 | -0.287 | **0.558**^†^ | *-0.405* | *-0.161* | -0.415 | 0.645^†^ | *-0.309* | 0.641^†^ | 0.116 | 0.038 | 0.0273 | *-0.531* | 0.658^†^ |
| Unemployment rate [-] | 0.344 | *-0.039* | 0.724^†^ | **-0.852**^‡^ | 0.283 | **0.687**^†^ | *0.338* | **-0.725**^†^ | *0.517* | **0.799**^†^ | -0.247 | *0.319* | *0.244* | -0.153 | -0.453 | -0.684^†^ |
| Youth unemployment [-] | 0.353 | 0.068 | 0.705^†^ | **-0.680**^†^ | 0.342 | *0.188* | -0.059 | **-0.793**^‡^ | **0.726**^†^ | *0.652* | -0.388 | *0.094* | *0.180* | -0.061 | -0.791^‡^ | -0.444 |
| Low income rate [-] | 0.071 | *-0.385* | 0.446 | *-0.142* | *-0.499* | *0.336* | -0.082 | *-0.133* | -0.217 | *0.499* | -0.571 | -0.585 | -0.487 | -0.420 | -0.202 | *0.161* |
| Average personal income [+] | -0.674^†^ | -0.231 | -0.481 | -0.194 | *0.003* | *-0.432* | *-0.025* | *0.087* | *-0.032* | **-0.650**^†^ | 0.209 | 0.188 | 0.290 | 0.586^†^ | 0.192 | 0.644^†^ |
| **Personal resources** |  |  |  |  |  |  |  |  |  |  |  |  |  |  |  |  |
| Life stress [-] | 0.256 | 0.436 | *-0.533* | 0.598^†^ | *-0.080* | -0.244 | -0.178 | *-0.223* | *0.071* | -0.533 | -0.232 | -0.068 | -0.384 | -0.254 | -0.515 | *0.305* |
| **Environmental factors** |  |  |  |  |  |  |  |  |  |  |  |  |  |  |  |  |
| Exposure to second-hand smoke [-] | 0.268 | 0.114 | 0.316 | *-0.453* | 0.167 | *0.495* | -0.386 | *-0.139* | -0.109 | *0.211* | -0.247 | **0.558**^†^ | *0.515* | -0.015 | *0.205* | -0.278 |
|  |  |  |  |  |  |  |  |  |  |  |  |  |  |  |  |  |
| ***Community and health system characteristics*** |  |  |  |  |  |  |  |  |  |  |  |  |  |  |  |  |
| **Community**^¥^ |  |  |  |  |  |  |  |  |  |  |  |  |  |  |  |  |
| Population [+/-] | 0.032 | 0.183 | -0.303 | 0.634^†^ | -0.378 | 0.000 | 0.189 | -0.086 | -0.007 | -0.204 | -0.214 | -0.439 | -0.611^†^ | -0.226 | -0.325 | 0.072 |
| Elderly population[+/-] | 0.778^‡^ | 0.670^†^ | -0.253 | 0.793^‡^ | 0.184 | -0.101 | 0.259 | -0.487 | 0.404 | 0.423 | -0.382 | -0.210 | -0.599^†^ | -0.633^†^ | -0.840^‡^ | -0.238 |
| Dependency ratio [-] | *-0.078* | *-0.491* | 0.017 | 0.610^†^ | **-0.563**^†^ | -0.280 | -0.008 | 0.743^‡^ | -0.824^‡^ | *0.102* | -0.454 | -0.205 | *0.066* | -0.093 | **0.656**^†^ | -0.421 |
| Urban population [+/-] | 0.009 | 0.240 | -0.629^†^ | 0.608^†^ | -0.120 | -0.224 | -0.114 | -0.283 | 0.204 | -0.473 | -0.132 | -0.303 | -0.571^†^ | -0.071 | -0.592^†^ | 0.434 |
| **Health System** |  |  |  |  |  |  |  |  |  |  |  |  |  |  |  |  |
| Hip replacement [-] | *-0.118* | 0.135 | **-0.728**^‡^ | 0.304 | 0.331 | -0.637^†^ | -0.006 | *-0.197* | -0.006 | -0.523 | *0.227* | *0.014* | *0.032* | *0.159* | -0.123 | *0.181* |
| Knee replacement [-] | **-0.562**^†^ | **-0.681**^†^ | *-0.047* | *-0.082* | *-0.277* | -0.457 | *0.159* | 0.724^‡^ | -0.694^‡^ | -0.478 | *0.003* | -0.057 | *0.369* | *0.352* | **0.827**^‡^ | -0.124 |
| Hysterectomy [-] | 0.657^†^ | 0.692^‡^ | 0.205 | *-0.016* | 0.569^†^ | *0.233* | *0.251* | *-0.542* | **0.604**^†^ | *0.404* | *0.061* | *0.104* | -0.194 | -0.453 | -0.632^†^ | -0.315 |
| Bypass surgery [-] | 0.448 | 0.252 | 0.293 | 0.103 | 0.165 | 0.494 | -0.208 | *-0.189* | *0.160* | *0.582* | -0.038 | -0.427 | -0.424 | -0.608^†^ | -0.359 | -0.381 |
| **Resources** |  |  |  |  |  |  |  |  |  |  |  |  |  |  |  |  |
| Total health expenditure per capita [+] | -0.678^†^ | -0.670^†^ | *0.375* | -0.800^‡^ | -0.313 | 0.285 | 0.290 | **0.730**^‡^ | **-0.565**^†^ | *-0.370* | 0.775^‡^ | *-0.008* | 0.470 | 0.546 | 0.912^‡^ | 0.098 |
| Public sector health expenditure per capita [+] | -0.716^‡^ | -0.585^†^ | *0.325* | -0.833^‡^ | -0.131 | 0.285 | 0.262 | **0.579**^†^ | *-0.419* | *-0.363* | 0.642^†^ | 0.205 | 0.648^†^ | 0.615^†^ | 0.851^‡^ | 0.248 |
| General/family physicians [+] | *0.062* | *0.522* | -0126 | -0.298 | *0.535* | *-0.010* | *-0.446* | -0.406 | 0.612^†^ | *-0.227* | 0.810^‡^ | *-0.076* | *-0.186* | *-0.095* | **-0.650**^†^ | 0.619^†^ |
| Certified specialists [+] | *0.536* | *0.401* | -0.377 | **0.781**^‡^ | -0.049 | 0.008 | 0.041 | -0.476 | 0.297 | 0.357 | **-0.642**^†^ | *-0.319* | **-0.674**^†^ | **-0.675**^†^ | **-0.738**^‡^ | *-0.145* |
| Registered nurses [+] | *0.010* | -0.149 | **0.569**^†^ | -0.824^‡^ | *0.308* | 0.519 | 0.033 | -0.153 | 0.023 | 0.339 | *-0.074* | 0.665^†^ | 0.756^‡^ | 0.270 | 0.403 | *-0.270* |
| Licensed practical nurses [+] | *0.542* | *0.357* | **0.649**^†^ | -0.312 | *0.424* | 0.570 | 0.136 | -0.585^†^ | 0.479 | 0.766^‡^ | *-0.146* | 0.210 | 0.109 | *-0.235* | *-0.254* | **-0.682**^†^ |
| Pharmacists [+] | *0.455* | **0.709**^‡^ | -0.410 | -0.181 | **0.743**‡ | *-0.086* | *-0.586* | -0.610^†^ | 0.663^†^ | 0.129 | 0.363 | 0.292 | *-0.004* | *-0.024* | **-0.798**^‡^ | 0.260 |
| Total physicians [+] | *0.408* | **0.588**^†^ | -0.337 | *0.508* | *0.284* | 0.004 | *-0.136* | -0.573^†^ | 0.572^†^ | 0.157 | 0.089 | *-0.268* | **-0.579**^†^ | **-0.712**^‡^ | **-0.901**^‡^ | 0.271 |
|  |  |  |  |  |  |  |  |  |  |  |  |  |  |  |  |  |

**^§^Dimensions and indicators of healthcare performance:**

**Acceptability:** Satisfied with family doctor (A); Satisfied with health care services (B); Satisfied with community health care (C)

**Accessibility:** Screening mammography (D); Pap smear (E); Difficulties accessing routine care (F); Difficulties accessing health information (G)

**Appropriateness:** Vaginal birth after Caesarean section (H); Caesarean sections (I)

**Effectiveness:** In-hospital 30-day stroke mortality (J); Pneumonia readmission rate (K); Ambulatory care sensitive conditions (L); Pneumonia & influenza hospitalizations (M)

**Safety:** Hip fracture hospitalizations (N)

**Other: health surveillance:** Chlamydia (O); Hepatitis C (P)

[-] implies that lower levels of the indicator are preferred; [+] implies that higher levels of the indicator are preferred

^†^ P < 0.05

^‡^ P < 0.01

**Bold**: correlation is significant in the possibly preferred direction and exceeds the critical level necessary for the sample size

*Italicized*: correlation is in the possibly preferred direction but is *not* significant at the critical level necessary for the sample size

^¥^ Only the dependency ratio indicator is assessed here using the decision rule since the other indicators could be preferred either way depending on the goal and audience
